# Transactions of the Thirty-sixth Session Illinois State Medical Society

**Published:** 1887-05

**Authors:** 


TRANSACTIONS OF THE THIRTY-SIXTH ANNUAL MEETING OF THE ILLINOIS STATE MEDICAL SOCIETY. *1886.*

This volume contains 297 pages, and includes the usual lists of officers of the society, together with the addresses delivered and the reports and papers read at the meeting, May 18th and 19th, 1886. The proceedings consist almost wholly of the reports of the various standing and special committees.

